# Innovative Application of Phytochemicals from Fermented Legumes and Spices/Herbs Added in Extruded Snacks

**DOI:** 10.3390/nu13124538

**Published:** 2021-12-17

**Authors:** Krystyna Szymandera-Buszka, Małgorzata Gumienna, Anna Jędrusek-Golińska, Katarzyna Waszkowiak, Marzanna Hęś, Artur Szwengiel, Anna Gramza-Michałowska

**Affiliations:** 1 Department of Gastronomy Science and Functional Foods, Faculty of Food Science and Nutrition, Poznań University of Life Sciences, Wojska Polskiego 31, 61-624 Poznan, Poland; krystyna.szymandera_buszka@up.poznan.pl (K.S.-B.); katarzyna.waszkowiak@up.poznan.pl (K.W.); marzanna.hes@up.poznan.pl (M.H.); anna.gramza@up.poznan.pl (A.G.-M.); 2 Department of Food Technology of Plant Origin, Faculty of Food Science and Nutrition, Poznań University of Life Sciences, Wojska Polskiego 31, 61-624 Poznan, Poland; malgorzata.gumienna@up.poznan.pl (M.G.); artur.szwengiel@up.poznan.pl (A.S.)

**Keywords:** legumes, fermented beans, extruded snacks, spices, sensory analysis, antioxidant activity

## Abstract

A trend related to adding legume seeds to various products has been observed. This work aimed to use fermented red bean/broad bean seeds and their hulls to produce extruded snacks with more beneficial nutritional properties and good sensory quality. Extruded snacks containing fermented ground seeds (50%) or hull (10%) of red bean/broad bean and corn grits with the addition of selected herbs/spices (0.5%) were prepared. The chemical composition, phenolic profile, antioxidant activity, and sensory quality were analysed. The results showed that the protein content ranged from 9 to 22.9 g 100 g^−1^, phenolic compounds ranged from 3.97 to 12.80 mg 100 g^−1^ (with the addition of herbs/spices, even up to 62.88 mg 100 g^−1^), and antioxidant activities ranged from 4.32 to 10.23 Trolox g^−1^ (ABTS assay), depending on the type of fermented materials. The addition of ground seeds/hull did not influence the consumer desirability, whereas the addition of selected herbs/spices, particularly lovage, increased it. The application of fermented red bean and broad bean seeds and their hulls, as part of the assumptions of the planetary diet, enabled enrichment of extruded corn products, which are often consumed by vegans and vegetarians, with nutritionally valuable ingredients.

## 1. Introduction

Legumes are a rich source of nutrients such as protein, low glycemic carbohydrates, and minerals [1,2,3]. Their recommended intake, which should be approximately 50 g/person/day (0–100 g/person/day), is not achieved in most countries. It is estimated to be approximately 21 g/day worldwide [4], approximately 4 g/person/day in Australia [3], while data on legume consumption in Poland indicate that it is 0.9 kg/year/person, which amounts to approximately 2.5 g/person/day [5].

Meanwhile, in the face of growing problems of excessive burdening of the environment by agricultural production, especially animal production, and the problem of malnutrition, which has not been solved for years, both in quantitative and qualitative terms, an increasing emphasis is put on the consumption of legumes. According to the assumptions of the so-called planetary health diet that is supposed to improve health, including reduced risk of coronary artery disease [6] and weight control [7], environmental benefits and also food security, the Food and Agriculture Organization of the United Nations (FAO), and other authors postulate that by 2050, global consumption of fruits, vegetables, nuts, and legumes will have to double, while the consumption of red meat and sugar must be reduced by over 50% [1,4,8,9]. In addition to the recommendations to increase the consumption of legumes, there is also a trend of adding them to various products. For example, the work on the nutritional and technological assessment of durum wheat flours enriched with faba bean flours and its use in bread production have been under-taken in Morocco. Such bread with a 40% substitution level showed good sensory quality, with a significantly higher amount of ash, proteins, mineral substances, total phenolic compounds and flavonoids, condensed tannins, and antiradical activity values [10]. Bouhlal et al. [11] showed a significant increase of nutritional quality parameters such as ash, proteins, fat, and energy, as well as of the total polyphenols, flavonoid content, and antiradical activity in four wheat–faba bean-enriched flours. In contrast, Ni et al. [12] showed that the bean hull, a valuable source of dietary fibre in bread, could be used to replace up to 21% of wheat flour without significantly impacting bread texture and volume. Moreover, Fendri et al. [13] revealed that fibre obtained from broad bean pods can be used to improve dough growth and to enhance the textural profile of bread.

However, legume seeds have some anti-nutrients, including trypsin and chymotrypsin inhibitors, which may negatively affect consumers, especially in view of the postulated increasing consumption of these products. Therefore, it seems crucial to subject legume seeds (as a whole or their components) to various technological processes, including fermentation, to reduce oligosaccharide content, but also to generate the formation of functional components such as vitamins (especially B2), antioxidants [14]. Back-slopping fermentation was applied, i.e., to mung bean (*Vigna radiata* (L.) Wilczek) flour, which resulted in better improvement of nutritional values than spontaneous methods; it was stated that this method could be encouraged at community levels [15]. A study has also been undertaken on the replacement of soy flour by fermented and unfermented faba bean flour in the production of gluten-free bread. It was shown that flour fermentation resulted in significant increases in Essential Amino Acid and Biological Value indexes compared to bread made from unfermented flour and soy flour without changing sensory properties [16].

Recent studies also indicate that legume flour can be incorporated into extruded snacks instead of cereals, significantly improving their functional properties as well as their nutritional value [17,18,19]. The cereal raw material most commonly used to make snacks is corn groat. It provides a good base for the development of corn-based food ingredients for the preparation of nutritious food products enriched with health-promoting ingredients [20]. Previous studies showed the possibility of enhancing their nutritional value by adding legumes [21]. It is also important to increase the functional properties of such products by adding the hull of these seeds. In legume seed production, it is still largely a by-product [22,23]. Therefore, using it as an additive for extruded snacks would constitute a use of waste and, at the same time, an increase in the nutritional value of the product [24]. This is especially true for fermented foods. Red beans and broad beans are often recommended in vegetarian diets [25,26]. Consumption of red kidney beans and broad beans has been linked disease reduction such diabetes, cancer, obesity, and coronary heart diseases. These products are characterised by interesting nutritional properties: high levels of complex proteins, carbohydrates, and dietary fibre, coupled with a low content of saturated lipids and the presence of bioactive compounds, such as polyphenols [26,27,28]. The addition of them to extruded snacks together with popular spices and herbs such as thyme, marjoram, oregano, basil, and lovage can diversify taste of final products and increase the content of phenolic compounds in them. The potential health benefits of their consumption are related to antioxidants activity, strengthening the immune system, antimicrobial activity, and anticancer effects [29,30,31]. However, their sensory properties still need to be refined to meet the expectations of increasingly demanding consumers [18].

Therefore, the aim of this study was to use fermented red bean and broad bean seeds as well as their hulls to produce extruded snacks in order to obtain their better nutritional values and desired sensory properties. In order to obtain this, in addition to fermented seeds or hulls, we also added selected spices (thyme, oregano, basil, and lovage) to corn crisps, which not only enriched the product with bioactive elements but also improved their taste and aroma. 

## 2. Materials and Methods

### 2.1. Material

Broad bean (*Vicia faba* L., cultivar Bachus) or red bean (*Phaseolus vulgaris* L., cultivar Red Kidney) constituted the sources of the legume seeds. Raw materials (red beans, broad beans) were obtained from Polish crops and purchased at the Seed Headquarters in Nochowo, Poland. Dried leaves of thyme (*Thymus vulgaris* L., cultivar Słoneczko), oregano (*Origanum vulgare* L.), basil (*Ocimum basilicum* L., cultivar Kasia), and lovage (*Levisticum officinale*, cultivar Amor) were purchased from a local herb shop.

### 2.2. Methods

#### 2.2.1. The Course of the Fermentation Process 

Legume seeds were subjected to the process of bacterial fermentation (*Lactobacillus plantarum* ATCC 8014). The raw material, after grinding and moistening to 55% humidity, was fermented at 37 °C for 18 h [32]. The obtained product was then dried in an air dryer (Memmert, Büchenbach, Germany) at 50 °C until the final moisture content was 15–17%. The semi-finished product prepared in this way was a component for the construction of solid products—extruded (crisps). 

#### 2.2.2. Preparation of Extruded Products

On the basis of preliminary trial sensory of texture (Ranking Test among 40 consumers to evaluate the amount of red and broad bean’s added—10, 20, 30, 40, 50, and 60%), we adopted the following variants for further research:

- corn grits (49.5%); 50% ground broad bean or ground red bean seeds after fermentation and 0.5% of herbs/species;
- corn grits (89.5%); 10% ground broad bean hull or ground red bean hull after fermentation and 0.5% herbs/species.

The shredded fermented raw material was mixed with spices or herbs (0.5%) and corn grits and then subjected to the extrusion process. The process of lactic fermentation was carried out at 37 °C for 18 h by *Lactobacillus plantarum* ATCC 8014. The obtained intermediate was dried to a final moisture content of 15–17% dm. The semi-finished product prepared in this way was a component for the construction of solid products—snacks. The extrusion was carried out in a single-screw extruder (type S-45-Metalchem, Gliwice, Poland). The process parameters were established in earlier studies by Gumienna et al. [33].

#### 2.2.3. Chemical Composition and Protein Digestibility

Chemical composition of the extruded samples was analysed, which included: dry mater content [34], ash content [35], and total protein content [36], as well as the reducing sugars with DNS (3,5-dinitrosalicylic acid), according to the Miller method [37], and soluble protein content by the Lowry method [38].

Protein digestibility was determined using in vitro pepsin-pancreatin method according to Saunders et al. [39]. Briefly, water and pepsin dissolved in 0.1 N HCl were added to the sample, and it was placed in a thermostat at 37 °C for 3 h. After that, it was neutralised, pancreatin was dissolved in phosphate buffer at pH = 8 containing 0.005 M, sodium azide was added, and the mixture was again thermostated at 37 °C for 24 h. After complete hydrolysis, 45% TCA was added to precipitate proteins, and the mixture was centrifuged at 5500× *g*. The protein content in the decanted liquid was determined [36] and expressed as percent of total protein content (% of protein digestibility).

#### 2.2.4. Preparation of Samples for the Analysis of Biologically Active Compounds and Their Determination

The sample was ground in a laboratory mill (Witko, Łódź, Poland). A total of 0.5 g of the homogeneous sample was weighed into a centrifuge tube and 10 mL of a 70:30 acetone/water extraction mixture [32]. The extraction mixture used in the research was applied on the basis of the research by Remiszewski et al. [40] and Gumienna et al. [41]. It was shaken for 60 min on a rocker shaker and centrifuged at 4125× *g* for 7 min. The supernatant fluid (extract) was decanted and used to perform the following analyses.

Tannin content: Determination of the amount of tannin was carried out using the reaction with a vanillin reagent [40]. The analysis was performed spectrophotometrically at a wavelength of λ = 500 nm. The content of tannins in the product was expressed as catechin equivalent (mg g^−1^ dm) (Sigma-Aldrich, Munich, Germany). 

Compounds that reduce the Folin–Ciocoltau reagent: The determination was carried out according to the spectrophotometric method with Folin–Ciocoltau reagent described by Singelton and Rossi [42] and modified by Remiszewski et al. [40]. The result was expressed as mg gallic acid equivalent (GAE g^−1^ dm) (Sigma-Aldrich, Munich, Germany).

Antiradical activity (ABTS assay): The antiradical activity was determined against the ABTS reagent (2,2′-azinobis-(3-ethylbenzothiazoline-6-sulphonic acid) (Sigma-Aldrich, Munich, Germany), according to the method described by Re et al. [43] as modified by Remiszewski et al. [40]. The analysis was performed spectrophotometrically at the wavelength λ = 735 nm by using a spectrophotometer (Biogenet, Józefów, Poland). Results of the ABTS assay were expressed as the capability of antioxidants to scavenge ABTS radicals relative to that of Trolox (a water-soluble vitamin E analogue) and reported as mg Trolox g^−1^ dm (Sigma-Aldrich, Munich, Germany). 

#### 2.2.5. Quantitative and Qualitative Determination of Phenolic Compounds by HPLC

Quantitative and qualitative determination of phenolic compounds was analysed by HPLC method, as described by Hertog et al. [44] and modified by Remiszewski et al. [40]. For the determination of phenolic compounds, the extract obtained earlier was subjected to acid hydrolysis in the presence of 6 N HCl (Sigma-Aldrich, Munich, Germany); then, the mixture was incubated at 90 °C for 2 h. The samples prepared in this way were filtered through filters with a diameter of 0.45 µm (Millipore) and subjected to chromatographic analysis. Separation of the compound mixture was carried out on an X-Terra C18 RP column (150 × 3.9 mm i.d. 5 μm) from Waters at 30 °C. The injection volume was 10 µL, and the flow rate of the mobile phase was 1 mL/min. The eluted fractions were monitored by UV–VIS detector (Waters Alliance, Taunton, MA, USA). The measurement time was 50 min. Formic acid (>98%), methanol (99%), and water were used as mobile phase (Sigma-Aldrich, Munich, Germany). A linear concentration gradient from 5% to 60% methanol was applied with a constant concentration of 5% formic acid. The recording of the chromatographic spectra was carried out in the wavelength range from 240–520 nm. Compounds were identified on the basis of spectra and retention times comparable to the standards (Sigma-Aldrich, Munich, Germany).

#### 2.2.6. Sensory Analysis 

Sensory Analysis was conducted in an appropriately designed and equipped laboratory of sensory analysis [45] at the Department of Gastronomy Science and Functional Foods, Poznan University of Life Sciences, Poland. The samples were coded with three-digit numbers, and the serving order of samples were random (program ANALSENS—v.5.0; Sopot; Poland, was used for coding and arrangement of serving order). 

The 30 g samples were served in plastic containers (150 mL), which were covered with lids. Unsweetened black tea (temperature of ≈45 °C) was used as a taste neutraliser between the samples.

The sensory profiling of taste was conducted by a 10-member trained panel [46,47]. A total of 8 descriptors were adapted for taste (essential oil, herbal, sour, salty, starch, broth, bitter, and strange) and 8 descriptors for aroma (essential oil, herbal, sour, starch, broth, bitter, lemon, and strange). The intensity of each score was determined using a 10 cm linear scale with appropriate margin descriptions. For attributes, uniform margin denotations were applied: “undetectable to very intensive”. All samples were assessed in two independent replications.

Consumer traits were conducted on a group of people 380 aged 21–56 (who consume corn snacks and legumes at least twice a month). The study was carried out in accordance with The Code of Ethics of the World Medical Association (consent no. 757/13). Women constituted 62% of the population analysed. All subjects gave written informed consent to participate. In consumer examinations, a 10 cm hedonic graphic scale was applied, with the following margin denotations: undesirable to highly desirable. Consumers evaluated desirability of colour, taste, aroma, texture, and overall desirability. All consumers rated all samples in one session (order of administration: 10 samples, 0.5 h interval, 10 samples, 0.5 h interval, and 12 samples).

### 2.3. Statistical Analysis

The results were analysed statistically with the STATISTICATM PL 13.3 software (StatSoft, Tulusa, OK, USA). The data were analysed for statistically significant differences with Tukey’s multiple range test (*p* = 0.05).

The active compounds content of the tested samples were analysed in 6 samples (2 independent samples and 3 measurements for each sample). For the overall evaluation of differences and similarities in sensory profiles, consumer analysis and content of active compounds of the tested samples the analysis of main components (PCA—principal component analysis) was used. Hypotheses were tested at α = 0.01. 

## 3. Results

### 3.1. Chemical Composition and Protein Digestibility of Extruded Products with the Addition of Fermented Bean Hulls or Ground Beans and Selected Herbs/Spices

All variants of extruded products were analysed for dry matter, ash, reducing substances, soluble protein, total protein, and digestibility of protein (Table 1). It was confirmed that total protein contents were higher in products with ground seeds added than hull—for red bean in the range of 60–80% and broad bean, 90–130%. Moreover, the analysis of these components pointed to higher total protein as well as soluble protein amount in the extruded snakes with added broad bean seeds when compared to red been seeds addition (20–34%). Taking into account the analysed spices, we found no effect on the amount of total and soluble protein. The higher amount of ash (for red bean in the range of 140–220% and broad bean to 140%), as well as reducing sugars (for red bean in the range of 117–180% and broad bean 64–130%), in products with added hulls was also confirmed. The results showed that protein digestibility of the tested extruded snakes was over 65 percent. 

### 3.2. Phenolic Acid and Flavonoid Amount in the Extruded Products with the Addition of Fermented Bean Hulls or Ground Beans and Selected Herbs/Spices

A qualitative and quantitative profile of phenolic compounds in the extruded products was shown (Table 2). A higher number of phenolic compounds was found in products with ground fermented beans (3.97 and 4.62 mg 100 g^−1^ dm) than in products with fermented bean hulls (11.17–12.8 mg 100 g^−1^ dm). In the extruded products with fermented bean hulls, as well as in those with ground fermented beans (controls), flavonoids such as myricetin, quercetin, luteolin, and kaempferol were identified. The products with ground fermented beans also had apigenin. Moreover, hydroxycinnamic acids were found in the extruded products with ground fermented red bean seeds and broad bean seeds, i.e., caffeic acid and ferulic acid, respectively.

The results of the study showed that the addition of herbs or spices (0.5%) significantly increased the content of phenolic compounds in the extruded products. The highest phenolic compound content was observed in all extruded products with lovage (42.03–62.88 mg 100 g^−1^ dm) (Table 2 and Table S1). In particular, the addition of lovage increased the content of the quercetin flavonoid, as well as hydroxycinnamic acids (mainly ferulic acid). The addition of oregano increased the content of phenolic compounds in the extruded products to 32.49–41.7 mg 100 g^−1^ dm. Luteolin and caffeic acid contributed the most to the phenolic compound profile of these products. In the products with 0.5% of basil and thyme, the contents of the determined phenolic compounds were 22.03–34.13 mg 100 g^−1^ dm (caffeic acid was dominant in the profile) and 17.21–19.5 mg 100 g^−1^ dm (luteolin was dominant).

### 3.3. Tannin Content and Antioxidant Activity of the Extruded Products with Added Fermented Bean Hulls or Ground Beans and Selected Herbs/Spices

Tannins are a class of polyphenolic molecules with molecular weights between 500 and 3000 Da. They act as an antinutrient compound of plant origin because they precipitate proteins, inhibit the digestive enzyme, and decrease the bioavailability of vitamins and minerals. However, tannins have also been considered as compounds with anticarcinogenic and antimutagenic potential, as well as antimicrobial properties [48,49]. Previous studies indicated the antioxidant and antiradical activity of tannins, as well as those extracted from legume seeds [50]. Among the control products (without added herbs/spices) tested in this study (Table 3), the lowest content of tannins (0.37 CAE mg g^−1^ dm) was found in the extruded product with fermented broad bean hull (10% added), and the highest content (1.64 CAE mg g^−1^ dm) in the extruded products with fermented ground red bean seeds (50% added). There were no clear trends regarding the effect of the addition of selected herbs/spices on the tannin amount in the extruded products (Table 3). The extruded products with the addition of fermented red bean were found to have more of tannins in the control sample (1.64 and 1.29 CAE mg g^−1^ dm in the extruded snacks with addition of ground seeds and hulls, respectively) than in the products where spices and herbs were added (0.76–1.32 CAE mg g^−1^ dm and 1.00–1.15 CAE mg g^−1^ dm). In the extruded snacks with broad bean added, there were more tannin in the samples with spices and herbs (1.42–1.61 CAE mg g^−1^ dm in the snacks with addition of ground seeds and 0.47–0.69 CAE mg g^−1^ dm in the snacks with hull addition) than in the control samples. The only exception were the snacks with addition of ground fermented broad bean seeds and oregano.

The Folin–Ciocalteu (FC) reaction is an antioxidant assay based on electron transfer, which measures the capacity to reduce FC reagent [51]. This study showed (Table 3) that a higher ability to reduce the Folin–Ciocolteu reagent was displayed by compounds extracted from the extruded products with the addition of fermented ground beans (3.23–3.35 mg GAE g^−1^ dm and 2.85–3.20 mg GAE g^−1^ dm) than from those where fermented hulls were added (2.18–2.83 mg GAE g^−1^ dm and 1.16–1.70 mg GAE g^−1^ dm). However, the addition of selected herbs/spices only slightly increased the results of FC assay.

As with the FC assay results, the ABTS test showed the effect of adding the fermented ground beans or hulls on the antiradical activity of the extruded products (Table 3). Higher antiradical activity was found for the extruded products with the addition of fermented ground beans (8.48–9.28 mg Trolox g^−1^ dm and 8.64–10.23 mg Trolox g^−1^ dm) than for those with the addition of fermented hulls (6.66–7.86 mg Trolox g^−1^ dm and 4.32–5.02 mg Trolox g^−1^ dm). Similarly to the results described before, adding herbs/spices slightly modified the antiradical activity of the extruded products with added fermented bean hulls/ground beans. There were no clear trends associated with the presence of individual spices in the products tested. Statistically (*p* > 0.05), among ground fermented red bean seeds and fermented red bean hull samples, a significantly higher antiradical activity was revealed for samples with basil and oregano, and basil and thyme, respectively, compared to the controls (without herbs/spices). For the extruded products with fermented broad beans (both hull and ground beans), the highest antiradical activity was demonstrated for samples with thyme (Table 3).

### 3.4. Sensory Quality of the Exruded Snacks with Fermented Broad Beans or Red Beans and Herb/Spices

The snacks tested can be targeted at many consumer groups, especially vegans. They aim to supplement a vegan diet with a product that has a higher nutritional value than traditional corn crisps. However, the success of a food product in the market is related to its sensory quality, especially consumer desirability. Therefore, this part of the presented experiment presented how the sensory characteristics of corn snacks with bean or broad bean hulls and herb/spices affect consumer desirability.

**Figure 1:**
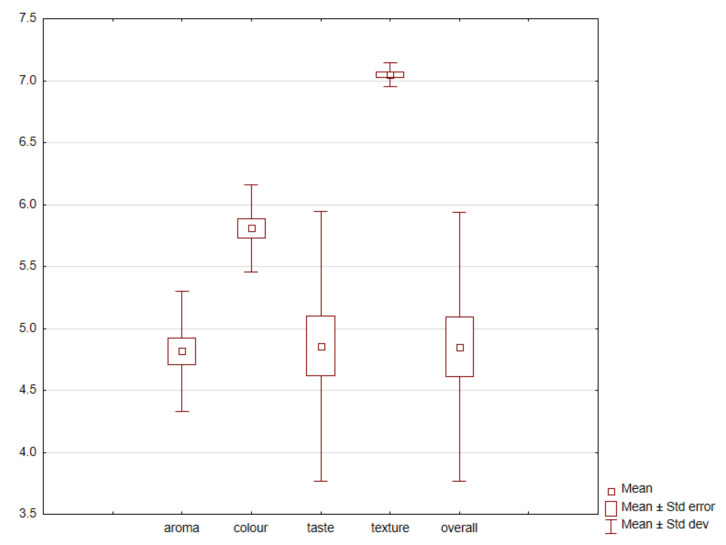
Box plot diagram of consumer desirability (colour, taste, aroma, texture, and overall desirability) of extruded snacks depending on the addition of fermented red or broad bean seeds/hulls and herbs/species (0.5%).

**Figure 2:**
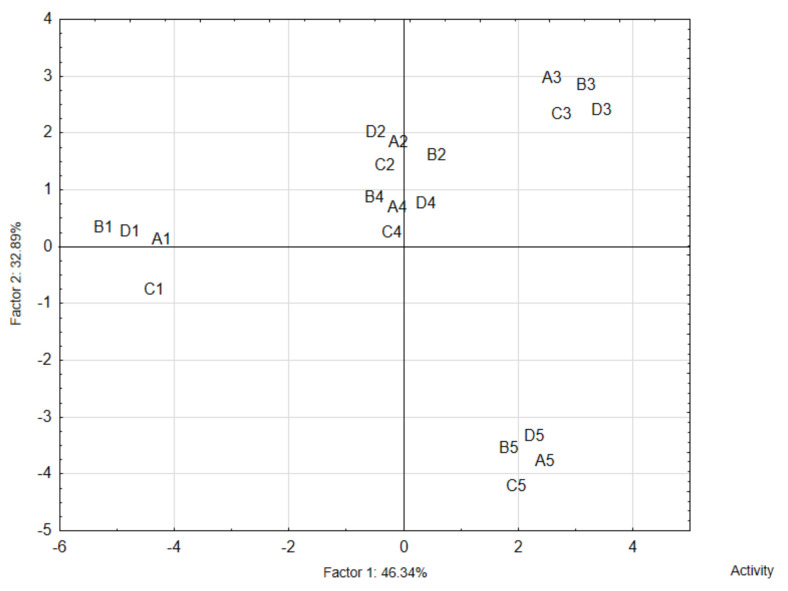
Principal component analysis (PCA) plots of data from overall desirability of extruded snacks with the addition of fermented red or broad bean seeds/hull and with herbs/species (0.5%), and without the addition of herbs/spices (Control); Addition of ground fermented red bean seeds (A): Control 1A, Thyme 2A, Oregano 3A, Basil 4A, Lovage 5A; Addition of fermented red bean hull (B): Control 1B, Thyme 2B, Oregano 3B, Basil 4B, Lovage 5B; Addition of ground fermented broad bean seeds (C): Control 1C, Thyme 2C, Oregano 3C, Basil 4C, Lovage 5C; Addition of fermented broad bean hull (D): Control 1D, Thyme 2D, Oregano 3D, Basil 4D; Lovage 5D.

Consumer evaluation results showed that the extruded snacks with fermented broad beans or red beans and spices had various overall desirability (Table S2 and Figure 1). Different taste and aroma desirability was also confirmed. The highest positive correlation was found between changes in overall desirability and taste desirability (*r* = 0.918). Samples with low taste desirability scores obtained low total desirability scores (Table S2 and Figure 2).

The overall desirability analysis showed that the products were rated between 3.7 and 6.8 points on a 10-point scale (Table S2). Similar rates were confirmed for taste desirability analysis (3.75–7.98).

It should be noted that the type of additive (i.e., fermented seeds or fermented hulls) did not cause differences in taste and overall desirability (Table S2 and Figure 2, *p* > 0.05). No statistically significant differences were identified in consumers’ ratings of these factors. However, a statistically significant effect of the herb/spice used on overall desirability was confirmed. The samples were placed in the PCA space (Figure 2), which demonstrated that they mainly focused on the spice variant, or its lack. 

The highest taste and overall desirability were identified for the products with lovage (7.75–7.92)—independently of type product. The samples without herb/spices had lower taste and overall desirability. The lowest results, especially taste desirability results, were identified for the samples with basil. 

In the sensory profiling of the tested extruded snacks, the perception of the following descriptors was defined and determined: taste (essential oil, herbal, sour, salty, starch, broth, bitter, and strange) and aroma (essential oil, herbal, sour, starch, broth, bitter, lemon, and strange). The results of the sensory profiling of these products are presented in Table S3.

Principal component analysis (PCA, Figure 2) was used to examine the relationship between the characteristic sensory profiles of the extruded snacks with fermented broad bean or red bean and herb/spices, and those without herb/spices —the control sample (consumer analysis variables)—and to identify the derived factors by which these variables can be classified. The PCA was also used to map the variants tested in this experiment (i.e., the samples with selected spices and legume seed variants) on these factors. The PCA showed that the first two factors (F1 and F2) were the most important elements of data variability (79%). The analysis revealed a significant dominant relationship between overall desirability and the taste descriptors (R = 0.91). Therefore, the taste descriptors were chosen to interpret the data. It is worth noting that the F1 factor was responsible for about 70% of taste variance.

**Figure 3:**
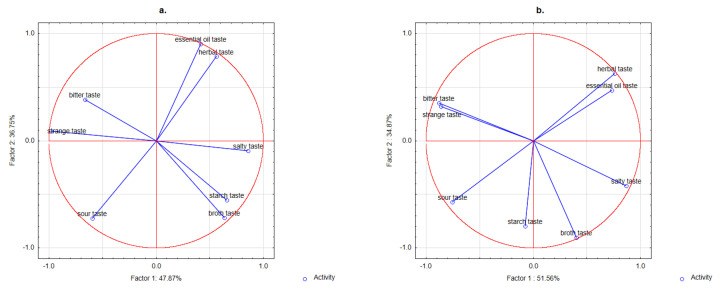

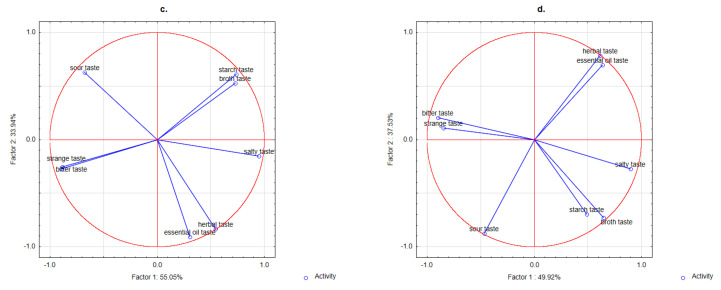
Principal component analysis (PCA) plots of data from sensory profiling of extruded snacks with addition of fermented red or broad beans/hull and with herbs/species (0.5%), and without the addition of herbs/spices (Control): (**a**) fermented ground red bean seeds 50%; (**b**) fermented red bean hull 10% panel; (**c**) fermented ground broad bean seeds 50%; (**d**) fermented broad bean hull 10%.

The analysis of sensory profiling did not confirm statistically significant differences between sample profilographs (types of descriptors and their intensities) depending on the type of legume variant added. On the other hand, a statistically significant effect of herb/spice on the type of descriptors and their intensity was confirmed. The samples were placed in the PCA space (Figure 3). The samples mainly focused on the spice variant or its lack.

For the taste descriptors, the projection of all variants on the factor-plane F1 × F2 (Figure 3) shows that samples with lovage and oregano were situated on the right of the F1 axis (i.e., they had positive coordinate values for F1). The flavour profile of the extruded snack with lovage, which had a low intensity of bitter and strange taste and a high intensity of broth taste, differed from the others to the largest extent. The samples with oregano showed similarities in profile to both lovage and other samples. A lower intensity of herbal, bitter, and strange taste (but higher than the samples with lovage) was confirmed. The samples with other spices (thyme, basil) and without them (control sample) were situated on the left (negative coordinate values for F1). Their taste profiles were characterised by a higher intensity of bitter and strange taste than the samples with lovage. The samples without spices differed from those with thyme, basil, and oregano in a lower intensity of essential oil and herbal taste.

### 3.5. Effect of Active Compounds of Spices on Sensory Desirability

Taste and overall desirability were found to be positively correlated with the intensity of broth taste (*r* = 0.923), starch taste (*r* = 0.714), salty taste (*r* = 0.758), and broth taste (*r* = 0.714) in all evaluated products (Table 4). A statistically negative correlation was also confirmed between overall and taste desirability and the intensity of bitter taste (*r* = −0.838) and strange taste (*r* = −0.712). The highest intensity of these two descriptors (bitter and strange) was demonstrated for corn crisps with thyme and basil. Previous results indicated that these descriptors and their intensities may be related to the presence of phenolic compounds. Statistical analysis confirmed the relationship between the content of some analysed ingredients and taste and overall desirability. A relationship was found between the intensity of selected taste descriptors and the content of analysed phenolic compounds.

A high correlation between the products with high overall desirability and with quercetin (QUE) (*r* = −0.960) was confirmed (Table 5). The analysis showed a high correlation between the amount of this flavonoid and the perceived intensity of broth taste (*r* = 0.971). There was also a correlation between the amount of QUE and the perceived intensity of salty taste (*r* = 0.744). The analysis showed a correlation between the amount of luteolin (LUT) and the perceived intensity of essential oil taste (*r* = 0.840) and herbal taste (*r* = 0.876). It was also demonstrated that the intensity of bitter taste was negatively correlated with the amount of QUE (*r* = −0.704). It is worth noting that the lower intensity of bitter taste in the samples with the highest amount of kaempferol (KEMP) was confirmed analytically. The largest content of this flavonoid was found in the samples with lovage. At the same time, these samples had the highest taste desirability and the lowest intensity of bitter taste. Statistical analysis confirmed a negative correlation between the content of this compound and the intensity of bitter taste (*r* = −0.636). These trends were confirmed for strange taste descriptor analysis. It was also confirmed that the higher the content of KEMP, the higher the strange taste reduction. There was a negative correlation concerning the amount of this compound (*r* = −0.616) but also concerning FA (ferulic acid; *r* = −0.689). 

## 4. Discussion

The results confirmed the possibility of using fermented seeds of broad beans or red beans in the production of extruded snacks. They contained flavonoids such as myricetin, quercetin, luteolin, and kaempferol. Products with added fermented hulls also had apigenin. Extruded snacks with fermented hulls had a lower number of phenolic compounds due to their lower content than the content of seeds (10% vs. 50%). 

A previous study [52] revealed that raw and cooked beans contain predominant quantities of flavonoids in their phenolic profiles, including quercetin, myricetin, cynidine, procyanidin, naringenin, catechin, hesperidin, and kaempferol, which is consistent with the results of this study. They found out that flavonoids were both in free and bound forms (as glucoside derivatives, mostly apigenin 7-O-glucoside, quercetin 3-O-glucoside, myricetin 3-O-glucoside, naringenin 7-O-glucoside, quercetin 4-O-galactoside, and kaempferol 3-O-glucoside). Variations in the flavonoids and their glucosides were observed depending on bean varieties. Kaempferol and its 3-O-glucosides were primarily found in pinto beans and diglucosides of kaempferol and quercetin in dark red kidney beans. 3-O-glucosides of malvidin, petunidin, and delphinidin were present in black beans; however, a small quantity of quercetin 3-O-glucoside and its malonates were found in light red kidney beans, whereas kaempferol monoglucoside. kaempferol 3-O-glucoside, and kaempferol 3-O-xylosyl glucoside were found in Italian beans. On the other hand, Brazilian beans contained non-glycosylated forms of isoflavonoids, including daidzein (0.82–12.91 mg 100 g^−1^) and genistein (0.26–0.97 mg 100 g^−1^). Black beans showed the highest concentrations of isoflavonoids among them, with daidzein being the main compound. The study [52] also demonstrated that raw and cooked beans contain phenolic acids, both benzoic acid derivatives (vanillic, *p*-hydroxybenzoic, and gallic acids) and those derived from cinnamic acid (ferulic, *p*-coumaric, and chlorogenic acids). Ferulic acid was the predominant phenolic acid in dry common beans. Studies also revealed demonstrated that cooking common beans at a high temperature does not change the content of phenolic acids. Raw common beans contained *p*-hydroxybenzoic acid (0.45–0.86 mg 100 g^−1^), vanillic acid (0.52–1.66 mg 100 g^−1^), coumaric acid (0.32–0.68 mg 100 g^−1^), and ferulic acid (0.17–0.36 mg 100 g^−1^).

The results of this study showed that adding 0.5% of selected herbs/spices significantly increased the content of flavonoids and phenolic acids in the extruded products. This is particularly true for lovage and oregano samples. The increase in the content of individual phenolic compounds in products with individual herbs/spices resulted from their participation in the phenolic profile of these additives. Slimestad et al. [53] reported that luteolin glucuronide together with rosmarinic acid were the main constituents of thyme. The amount of luteolin in thyme was determined to be 660 mg 100 g^−1^ on average, with the amount of luteolin in fresh herbs being higher (1.489 mg 100 g^−1^). Nieto [54] showed that methanolic extracts of thyme were the sources of flavonols, such as quercetin-7-O-glucoside, and phenolic acids (*p*-coumaric, caffeic, rosmarinic, cinnamic, carnosic, ferulic, quinic, and caeoylquinic acids), as well as flavanones (naringenin) and flavones (apigenin). Using other solvents such as butanol, ethyl acetate, and hexane, they extracted various compounds from thyme, including saponins, steroids, flavonoids, alkaloids, and tannins. In basil, the total amount of phenolic acid was determined to be 52.61 mg of gallic acid per gram of dry extract [55]. Phenolic acids detected in the basil extract were chlorogenic, *p*-hydroxybenzoic, caffeic, vanillic, rosmarinic, and cinnamic acids. The most dominant acids were rosmarinic (0.18 mg g^−1^ of dry extract) and cinnamic (0.18 mg g^−1^ of dry extract). The total flavonoid amount reported by Teofilovic at al. [55] was 0.52 mg of quercetin per gram of dry extract, and among flavonoid components, quercetin (4.77 mg g^−1^ of dry extract) and naringenin (0.18 mg g^−1^ of dry extract). Rosmarinic acid, apigenin, luteolin, quercetin, scutellarein, and their derivatives were identified as the major phenolic compounds in oregano; however. the content and distribution of the phenolic compound in oregano varied depending on cultivar, geographical factors, and environmental factors [56]. Nour et al. [57] analysed the phenolic profile and antiradical activity of culinary herbs (i.e., parsley, dill, lovage, and celery leaves). They found that lovage had the highest total amount of phenolic compound (577.04 mg GAE 100 g^−1^ dm) and flavonoids (298.38 mg QE 100 g^−1^), and the highest antioxidant activity (1462.52 mg Trolox 100 g^−1^) among the selected herbs, demonstrating its outstanding value in terms of antioxidant activity and content of bioactive compounds. In the phenolic profile of lovage, the main compounds were quercetin, myricetin, rutin, ellagic acid, and sinapic acid. In line with the results presented in this study, Kozłowska et al. [58] also identified neochlorogenic acid in the phenolic profile of a lovage extract.

This study showed that a higher ability to reduce the Folin–Ciocolteu reagent was demonstrated by compounds extracted from the extruded products with the addition of fermented ground beans than from those where fermented hulls were added (Table 3). The FC assay has been widely applied in the determination of the total phenol/polyphenol content of plant-derived food and biological samples; however, the method is not specific only for phenolic compounds because it is based on the redox reaction [51,59], and the results can be affected by various interfering substances present in crude plant extracts (e.g., sugars, proteins, and other non-phenolic compounds). This non-specificity of the assay may explain the higher values determined in this study for products with the addition of ground fermented beans compared to those where fermented bean hulls were added, independently of herb/spice additions. Clarification of this problem requires furthers research.

The analysis of the sensory analysis results confirmed a high texture and colour desirability of all samples, regardless of the additive used. Previous data in some cases indicated potential texture deterioration in such products when legumes are added [18]. Similarly, previously concerns about the addition of spices (especially thyme and basil) and colour deterioration in consumer assessment are not confirmed in this study [17]. In contrast, a highly significant impact of taste on overall desirability was confirmed. In this study, lower taste desirability was found, and thus overall desirability among the samples with a high intensity of bitter taste. This is supported by previous studies indicating low acceptance of this descriptor among consumers. Previous studies also revealed that bitter taste can reduce consumer acceptance of food products [60,61,62,63]. The highest statistically significant taste and overall desirability were found among the samples with lovage regardless of the addition of legume seeds. 

The profile analysis showed the highest intensity of essential oil taste and herbal taste in the samples containing thyme, basil, and oregano. A negative correlation between taste desirability and these descriptors was confirmed. These trends confirm previous data showing restrictions on the use of spices in food production due to the presence of these descriptors [64].

The taste profile analysis also indicated the highest perceived intensity of bitter taste in the samples with thyme and basil, which confirms previous studies on the addition of these spices to meat products [65,66]. Previous literature [65] indicates a correlation between the presence of bitter taste in the product with the presence of kaempferol and quercetin [60,67]. The results presented in this paper did not confirm this trend. The intensity of this taste was found to be the lowest in the samples with the highest concentration of kaempferol and quercetin. This was true for all samples with lovage. Consumer analysis confirmed that taste and overall desirability increased when the intensity of broth taste went up. This may indicate that the broth-like smell and lovage taste, which are due to the presence of terpene-like compounds, mask the bitter taste. A relatively high content of these compounds in lovage noticeably reduced the intensity of the bitter taste. However, this initial hypothesis should be confirmed/verified in subsequent studies.

## 5. Conclusions

This study indicates the possibility of using fermented red bean and broad bean seeds and by-products in the form of their hulls for the production of extruded snacks. Their addition increases the common nutritional value of these snacks, measured by the content of mainly protein and phenolic compounds with proven health-promoting effects in numerous studies. Adding selected herbs, especially lovage, positively affects the sensory profile and consumer desirability of these products. The use of fermented seeds of red beans and broad beans, as well as their hulls, is not only in line with the principles of the planetary health diet but also serves as a nutritionally valuable enrichment of corn products often consumed by vegans and vegetarians.

In future studies, it is worthwhile to further develop this topic by studying for example interactions between corn product components as well as the antioxidant properties of the new products tested to determine their usefulness in the diet of people of today more precisely.

## Figures and Tables

**Table 1:** Chemical composition and protein digestibility of extruded snacks with the addition of fermented red or broad bean seeds/hulls and with herbs/species (0.5%), and the controls (without herbs/spices addition).

Products	Dry Matter (mg g^−1^)	Ash (mg g^−1^)	Reducing Substance (mg g^−1^ dm)	Soluble Protein (mg g^−1^ dm)	Total Protein (mg g^−1^ dm)	Protein Digestibility (%)
Extruded snacks with addition of ground fermented red bean seeds (50%)						
Control	955.50 ± 2.37 ^d^	24.56 ± 0.13 ^a^	17.01 ± 0.29 ^c^	6.03 ± 0.55 ^a^	173.81 ± 4.72 ^c^	66.69 ± 3.66 ^a^
Lovage	947.44 ± 1.27 ^b^	25.38 ± 1.00 ^b^	16.32 ± 0.11 ^b^	7.75 ± 0.18 ^b^	174.10 ± 3.36 ^c^	65.86 ± 0.58 ^a^
Basil	949.50 ± 2.38 ^c^	26.76 ± 1.44 ^c^	15.80 ± 0.31 ^a^	7.74 ± 0.51 ^b^	172.85 ± 2.10 ^b^	71.65 ± 0.99 ^b^
Oregano	943.54 ± 1.13 ^a^	26.71 ± 0.52 ^c^	16.95 ± 0.11 ^c^	6.97 ± 0.51 ^a^	175.23 ± 4.64 ^d,^	69.43 ± 1.36 ^b^
Thyme	948.54 ± 0.82 ^c^	26.01 ± 0.62 ^b^	15.95 ± 0.11 ^a^	6.27 ± 0.51 ^a^	170.23 ± 4.62 ^a^	69.29 ± 1.86 ^b^
Extruded snacks with addition of fermented red bean hull (10%)						
Control	946.54 ± 2.08 ^b^	10.44 ± 0.01 ^b^	5.97 ± 0.20 ^a^	10.91 ± 0.21 ^a^	103.14 ± 2.41 ^d^	84.75 ± 2.65 ^a^
Lovage	947.41 ± 1.15 ^b^	8.31 ± 0.02 ^a^	6.27 ± 0.09 ^v^	11.042 ± 0.14 ^a^	99.82 ± 0.66 ^b^	84.15 ± 3.81 ^a^
Basil	945.85 ± 0.51 ^b^	10.61 ± 0.20 ^b^	7.26 ± 0.01 ^d^	12.582 ± 0.25 ^c^	100.55 ± 1.51 ^c^	85.85 ± 3.82 ^a^
Oregano	947.45 ± 0.69 ^b^	8.25 ± 0.05 ^a^	6.57 ± 0.16 ^c^	12.00 ± 0.15 ^b^	100.23 ± 2.73 ^c^	83.84 ± 1.40 ^a^
Thyme	942.79 ± 0.86 ^a^	8.40 ± 0.10 ^a^	6.61 ± 0.19 ^c^	12.20 ± 0.10 ^b^	93.31 ± 3.72 ^a^	91.18 ± 1.23 ^b^
Extruded snacks with addition of ground fermented broad bean seeds (50%)						
Control	933.94 ± 1.12 ^b^	19.29 ± 1.29 ^a^	13.66 ± 0.11 ^b^	21.91 ± 0.43 ^c^	212.25 ± 2.20 ^b^	78.45 ± 0.69 ^c^
Lovage	934.95 ± 0.53 ^c^	19.40 ± 1.71 ^a^	12.72 ± 0.26 ^a^	17.32 ± 0.39 ^a^	219.05 ± 5.94 ^d^	70.77 ± 1.44 ^b^
Basil	928.75 ± 0.61 ^b^	24.46 ± 2.06 ^b^	13.15 ± 0.09 ^a^	19.37 ± 1.70 ^b^	217.34 ± 6.96 ^c^	65.82 ± 1.98 ^a^
Oregano	931.25 ± 0.51 ^b^	18.15 ± 1.20 ^a^	13.15 ± 0.07 ^a^	21.02 ± 0.50 ^c^	210.19 ± 4.96 ^a^	69.02 ± 1.68 ^b^
Thyme	923.40 ± 0.35 ^a^	22.65 ± 1.42 ^b^	15.15 ± 0.04 ^c^	18.04 ± 1.40 ^ba^	229.42 ± 15.99 ^c^	67.73 ± 2.53 ^ba^
Extruded snacks with addition of fermented broad bean hull (10%)						
Control	930.27 ± 0.52 ^a^	13.69 ± 1.18 ^c^	7.20 ± 0.21 ^b^	10.99 ± 0.22 ^a^	92.03 ± 5.58 ^a^	80.52 ± 1.69 ^a^
Lovage	939.63 ± 0.73 ^c^	9.30 ± 1.98 ^a^	6.95 ± 0.59 ^b^	15.98 ± 0.69 ^c^	111.96 ± 28.11 ^b^	87.77 ± 1.44 ^b^
Basil	940.25 ± 1.71 ^c^	10.50 ± 0.56 ^b^	7.98 ± 0.10 ^c^	14.85 ± 0.90 ^b^	103.19 ± 0.49 ^a^	89.82 ± 1.98 ^bc^
Oregano	939.71 ± 0.25 ^c^	18.80 ± 0.66 ^b^	6.71 ± 0.05 ^b^	14.60 ± 0.38 ^b^	114.12 ± 2.38 ^c^	90.03 ± 2.21 ^c^
Thyme	936.01 ± 0.22 ^a^	9.76 ± 0.33 ^a^	6.52 ± 0.04 ^a^	14.49 ± 0.30 ^b^	117.41 ± 6.48 ^c^	92.73 ± 2.53 ^c^

Different letters denote a significant difference for means (*n* = 6) for the same lines at a α ≤ 0.05.

**Table 2:** Phenolic compounds of extruded snacks with the addition of fermented red or broad bean seeds/hulls and with herbs/species (0.5%), and the controls (without herbs/spices addition).

Products	Phenolic Compounds [mg 100 g^−1^ dm]									
	NCA	CA	p-CA	FA	MYR	QUE	LUT	KEMP	API	Total
Extruded snacks with addition of ground fermented red bean seeds (50%)										
Control	nd	nd	nd	nd	0.02 ± 0.00 ^a^	2.67 ± 0.2 ^c^	0.02 ± 0.00 ^a^	1.93 ± 0.3 ^c^	nd	4.62
Lovage	0.60 ± 0.2 ^a^	1.41 ± 0.4 ^a^	2.46 ± 0.5 ^b^	9.21 ± 1.4 ^c^	0.63 ± 0.2 ^b^	34.97 ± 8.1 ^d^	0.13 ± 0.01 ^a^	4.9 ± 0.8 ^d^	nd	54.31
Basil	0.44 ± 0.2 ^a^	12.02 ± 2.6 ^c^	2.74 ± 0.6 ^b^	2.20 ± 0.8 ^b^	1.11 ± 0.3 ^b^	1.21 ± 0.1 ^a^	1.0 ± 0.2 ^b^	1.41 ± 0.1 ^b^	0.9 ± 0.3 ^a^	22.03
Oregano	0.41 ± 0.25 ^a^	6.01 ± 0.6 ^b^	1.91 ± 0.4 ^b^	0.79 ± 0.1 ^a^	nd	1.81 ± 0.3 ^b^	19.33 ± 5.1 ^d^	1.81 ± 0.2 ^c^	1.42 ± 0.5 ^a^	32.49
Thyme	nd	1.32 ± 0.3 ^a^	1.12 ± 0.2 ^a^	2.33 ± 0.7 ^b^	nd	1.11 ± 0.1 ^a^	10.23 ± 0.7 ^c^	1.09 ± 0.1 ^a^	1.01 ± 0.6 ^a^	17.21
Extruded snacks with addition of fermented red bean hull (10%)										
Control	nd	0.86 ± 0.03	nd	nd	0.05 ± 0.00 ^a^	3.98 ± 0.9 ^c^	0.7 ± 0.01 ^b^	4.43 ± 0.3 ^d^	1.15 ± 0.07 ^b^	11.17
Lovage	0.63 ± 0.2 ^a^	1.31 ± 0.15 ^a^	1.46 ± 0.5 ^b^	9.97 ± 1.4 ^c^	0.43 ± 0.1 ^c^	38.77 ± 9.1 ^d^	0.11 ± 0.00 ^a^	7.10 ± 1.3 ^e^	nd	59.78
Basil	0.46 ± 0.1 ^a^	20.12 ± 5.2 ^c^	1.94 ± 0.4 ^b^	2.79 ± 0.5 ^b^	0.67 ± 0.1 ^d^	1.82 ± 0.25 ^b^	0.21 ± 0.02 ^a^	1.11 ± 0.21 ^a^	0.7 ± 0.01 ^a^	28.82
Oregano	0.43 ± 0.1 ^a^	9.11 ± 2.2 ^b^	1.31 ± 0.3 ^b^	0.99 ± 0.01 ^a^	0.31 ± 0.2 ^c^	2.17 ± 0.15 ^b^	22.23 ± 4.5 ^d^	3.08 ± 0.48 ^c^	1.44 ± 0.1 ^c^	41.07
Thyme	nd	1.32 ± 0.2 ^a^	0.72 ± 0.2 ^a^	2.93 ± 0.5 ^b^	0.14 ± 0.01 ^b^	0.98 ± 0.11 ^a^	12.04 ± 0.8 ^c^	2.14 ± 0.1 ^b^	1.02 ± 0.2 ^b^	19.29
Extruded snacks with addition of ground fermented broad bean seeds (50%)										
Control	nd	0.82 ± 0.08 ^a^	nd	nd	nd	2.01 ± 0.25 ^c^	0.09 ± 0.00 a	1.05 ± 0.1 ^b^	nd	3.97
Lovage	0.3 ± 0.01 ^a^	2.41 ± 0.2 ^b^	2.21 ± 0.5 ^b^	6.31 ± 1.2 ^c^	0.41 ± 0.1 ^b^	25.37 ± 6.1 ^d^	1.12 ± 0.2 ^b^	3.9 ± 0.4 ^d^	nd	42.03
Basil	0.51 ± 0.11 ^b^	18.01 ± 4.2 ^d^	2.19 ± 0.3 ^b^	2.30 ± 0.2 ^b^	0.56 ± 0.08 ^b^	1.19 ± 0.1 ^b^	1.01 ± 0.1 ^b^	0.91 ± 0.01 ^a^	nd	26.68
Oregano	0.49 ± 0.1 ^b^	8.78 ± 1.2 ^c^	1.01 ± 0.19 ^a^	0.81 ± 0.2 ^a^	0.06 ± 0.00 ^a^	1.91 ± 0.2 ^c^	18.08 ± 4.4 ^d^	1.91 ± 0.2 ^c^	0.99 ± 0.15 ^a^	34.04
Thyme	nd	1.12 ± 0.2 ^a^	0.72 ± 0.1 ^a^	3.33 ± 1.0 ^b^	nd	0.18 ± 0.00 ^a^	11.09 ± 0.9 ^c^	1.08 ± 0.11 ^b^	0.8 ± 0.1 ^a^	18.23
Extruded snacks with addition of fermented broad bean hull (10%)										
Control	nd	2.13 ± 0.3 ^b^	nd	1.81 ± 0.8 ^b^	0.21 ± 0.02 ^a^	4.01 ± 0.79 ^c^	0.8 ± 0.02 ^b^	3.13 ± 0.2 ^b^	0.71 ± 0.06 ^a^	12.8
Lovage	0.42 ± 0.1 ^a^	1.01 ± 0.2 ^a^	1.66 ± 0.6 ^b^	9.98 ± 1.2 ^c^	0.43 ± 0.1 ^c^	40.17 ± 9.6 ^d^	0.09 ± 0.01 ^a^	9.12 ± 1.1 ^d^	nd	62.88
Basil	0.48 ± 0.1 ^a^	25.67 ± 6.3 ^d^	2.04 ± 0.35 ^b^	3.09 ± 0.5 ^b^	0.67 ± 0.2 ^c^	0.92 ± 0.17 ^a^	0.18 ± 0.01 ^a^	1.08 ± 0.08 ^a^	nd	34.13
Oregano	0.39 ± 0.1 ^a^	10.13 ± 1.3 ^c^	1.81 ± 0.4 ^b^	1.09 ± 0.3 ^a^	0.31 ± 0.02 ^b^	2.67 ± 0.3 ^b^	22.29 ± 4.5 ^d^	3.67 ± 0.2 ^c^	1.94 ± 0.9 ^b^	44.3
Thyme	nd	1.62 ± 0.3 ^b^	0.62 ± 0.01 ^a^	2.98 ± 0.6 ^b^	0.14 ± 0.01 ^a^	0.88 ± 0.09 ^a^	11.02 ± 0.3 ^c^	1.14 ± 0.03 ^a^	1.12 ± 0.4 ^b^	19.5

NCA—neochlorogenic acid, CA—caffeic acid, p-CA—*p*-coumaric acid, FA—ferulic acid, MYR—myricetin, QUE—quercetin, UT—luteolin, KEMP—kaempferol, API—apigenin, nd—not detected. Different letters denote a significant difference for means (*n* = 6) for the same lines at a α ≤ 0.05.

**Table 3:** Antioxidant activity and tannin content of extruded snacks with the addition of fermented red or broad beans/hull and with herbs/species (0.5%), and the controls (without herbs/spices addition).

Products	Folin–Ciocalteu Assay (FC) (mg GAE g^−1^ dm)	Antiradical Activity (ABTS) (mg Trolox g^−1^ dm)	Tannins (CAE mg g^−1^ dm)
Extruded snacks with addition of ground fermented red bean seeds (50%)			
Control	3.27 ± 0.04 ^a^	8.75 ± 0.21 ^a^	1.64 ± 0.03 ^e^
Lovage	3.23 ± 0.06 ^a^	8.55 ± 0.11 ^a^	1.32 ± 0.03 ^d^
Basil	3.33 ± 0.08 ^a^	9.13 ± 0.06 ^b^	1.13 ± 0.07 ^c^
Oregano	3.35 ± 0.03 ^a^	9.28 ± 0.05 ^c^	0.94 ± 0.01 ^b^
Thyme	3.32 ± 0.03 ^a^	8.48 ± 0.05 ^a^	0.76 ± 0.05 ^a^
Extruded snacks with addition of fermented red bean hull (10%)			
Control	2.18 ± 0.02 ^a^	6.66 ± 0.11 ^a^	1.29 ± 0.03 ^c^
Lovage	2.32 ± 0.03 ^b^	6.73 ± 0.38 ^a^	1.12 ± 0.05 ^b^
Basil	2.83 ± 0.12 ^e^	7.86 ± 0.42 ^b^	1.01 ± 0.02 ^a^
Oregano	2.43 ± 0.01 ^c^	6.83 ± 0.24 ^a^	1.00 ± 0.02 ^a^
Thyme	2.55 ± 0.07 ^d^	7.53 ± 0.02 ^b^	1.15 ± 0.08 ^b^
Extruded snacks with addition of ground fermented broad bean seeds (50%)			
Control	2.85 ± 0.02 ^a^	8.64 ± 0.28 ^a^	1.22 ± 0.05 ^b^
Lovage	2.99 ± 0.04 ^b^	9.48 ± 0.12 ^b^	1.61 ± 0.12 ^c^
Basil	3.01 ± 0.07 ^b^	9.66 ± 0.07 ^b^	1.42 ± 0.08 ^c^
Oregano	3.10 ± 0.04 ^b^	9.39 ± 0.14 ^b^	1.00 ± 0.08 ^a^
Thyme	3.20 ± 0.02 ^c^	10.23 ± 0.13 ^c^	1.60 ± 0.09 ^c^
Extruded snacks with addition of fermented broad bean hull (10%)			
Control	1.16 ± 0.01 ^a^	4.32 ± 0.07 ^a^	0.37 ± 0.01 ^a^
Lovage	1.33 ± 0.02 ^b^	4.89 ± 0.10 ^b^	0.47 ± 0.03 ^b^
Basil	1.57 ± 0.06 ^c^	4.74 ± 0.33 ^b^	0.49 ± 0.03 ^b^
Oregano	1.62 ± 0.05 ^c^	4.84 ± 0.34 ^b^	0.59 ± 0.02 ^c^
Thyme	1.70 ± 0.01 ^d^	5.02 ± 0.19 ^b^	0.69 ± 0.08 ^c^

CAE—catechin equivalent; GAE—gallic acid equivalent. Different letters denote a significant difference for means (*n* = 6) for the same lines at a α ≤ 0.05.

**Table 4:** Correlations coefficients between the intensity of descriptors and consumer analysis parameters of extruded snacks with the addition of fermented red or broad bean seeds/hull and with herbs/species (0.5%), and the controls (without herbs/spices addition).

	Aroma	Colour	Taste	Texture	Overall
Essential oil aroma	0.085	0.108	−0.227	−0.026	−0.248
Herbal aroma	0.181	0.165	−0.110	−0.066	−0.132
Starch aroma	−0.438	−0.322	−0.658	0.021	−0.671
Lemon aroma	0.399	0.197	−0.094	−0.069	−0.105
Bitter aroma	−0.412	−0.403	−0.731	0.046	−0.734
Strange aroma	−0.635	−0.500	−0.252	−0.021	−0.248
Sour aroma	−0.569	−0.260	−0.029	0.220	−0.023
Broth aroma	0.113	0.260	0.915	−0.016	0.923
Essential oil taste	0.046	0.085	−0.063	−0.064	−0.092
Herbal taste	0.203	0.233	−0.035	−0.043	−0.057
Sour taste	−0.408	−0.289	−0.012	0.069	0.001
Salty taste	−0.546	−0.145	0.769	−0.152	0.758
Broth taste	0.112	0.244	0.922	−0.017	0.932
Starch taste	0.267	0.201	0.721	−0.239	0.714
Bitter taste	0.116	−0.233	−0.838	−0.046	−0.825
Strange taste	−0.212	−0.296	−0.712	0.110	−0.707

**Table 5:** Correlations coefficients between sensory parameters and the content of phenolic compounds of extruded snacks with addition of fermented red bean or broad bean seeds/hulls and with herbs/species (0.5%), and the controls (without herbs/spices addition).

	NCA	CA	p-CA	FA	MYR	QUE	LUT	KEMP	API	Total
Aroma	0.536	0.647	0.614	0.164	0.594	−0.029	−0.069	−0.209	−0.188	0.259
Colour	0.320	0.103	0.235	0.197	0.163	0.191	0.065	0.113	−0.136	0.288
Taste	0.419	−0.430	0.287	0.856	0.140	0.960	−0.194	0.837	−0.416	0.773
Texture	0.011	−0.051	−0.189	−0.099	−0.035	0.017	0.013	0.130	0.054	−0.020
Overall	0.421	−0.411	0.291	0.863	0.155	0.970	−0.216	0.844	−0.433	0.776
Essential oil aroma	0.137	0.125	0.126	−0.353	−0.255	−0.398	0.971	−0.246	0.807	0.133
Herbal aroma	0.292	0.181	0.286	−0.223	−0.164	−0.275	0.932	−0.197	0.696	0.276
Starch aroma	−0.841	−0.321	−0.820	−0.730	−0.686	−0.723	0.320	−0.526	0.432	−0.824
Lemon aroma	0.500	0.424	0.561	0.006	0.219	−0.150	0.688	−0.092	0.612	0.435
Bitter aroma	−0.762	−0.174	−0.720	−0.752	−0.429	−0.725	0.074	−0.496	0.389	−0.870
Strange aroma	−0.567	−0.338	−0.655	−0.307	−0.315	−0.168	−0.277	0.087	0.101	−0.495
Sour aroma	−0.514	−0.445	−0.691	−0.253	−0.357	0.007	−0.475	0.118	−0.311	−0.497
Broth aroma	0.506	−0.223	0.407	0.945	0.304	0.956	−0.293	0.846	−0.477	0.835
Essential oil taste	0.335	0.171	0.241	−0.255	−0.219	−0.227	0.840	−0.156	0.620	0.261
Herbal taste	0.326	0.169	0.278	−0.153	−0.211	−0.195	0.876	−0.150	0.570	0.322
Sour taste	−0.351	−0.221	−0.431	−0.097	−0.074	0.062	−0.663	0.002	−0.520	−0.419
Salty taste	−0.089	−0.718	−0.126	0.630	−0.204	0.744	−0.203	0.719	−0.230	0.385
Broth taste	0.541	−0.176	0.454	0.940	0.401	0.971	−0.443	0.786	−0.586	0.789
Starch taste	0.744	0.118	0.764	0.703	0.439	0.675	0.094	0.507	−0.130	0.890
Bitter taste	−0.425	0.379	−0.219	−0.550	0.091	−0.704	−0.228	−0.636	0.144	−0.714
Strange taste	−0.479	0.132	−0.543	−0.689	−0.199	−0.616	−0.199	−0.404	0.141	−0.745

NCA—neochlorogenic acid, CA—caffeic acid, p-CA—*p*-coumaric acid, FA—ferulic acid, MYR—myricetin, QUE—quercetin, LUT—luteolin, KEMP—kaempferol, API—apigenin.

## Data Availability

The data are available as Supplementary Data.

## Supplementary Materials

The following are available online at www.mdpi.com/article/10.3390/nu13124538/s1, Table S1: Phenolic compounds of herb/spices added to extruded snacks. Table S2: Mean scores of consumer desirability of corn snacks with fermented broad bean or bean and herb/spices, and the control sample (without spices addition). Table S3: Mean scores (*n* = 17) of sensory taste and aroma profiling of corn snacks with fermented broad bean or bean and herb/spices, and the control sample (without herb/spices addition).
