# Supplementary material for: Innovative Application of Phytochemicals from Fermented Legumes and Spices/Herbs Added in Extruded Snacks
Source: Nutrients. 2021 Dec 17;13(12):4538. doi: 10.3390/nu13124538 (PMC8703383; doi:10.3390/nu13124538)
Supplement: Supplementary file 1 [file nutrients-13-04538-s001.zip › nutrients-1484650-supplementary.pdf]

Table S1. Phenolic compounds of herbs/spices added to extruded snacks.

Raw materials	Phenolic compounds [mg/100g d.m.]									
	**NCA**	**CA**	**p-CA**	**FA**	**MYR**	**QUE**	**LUT**	**KEMP**	**API**	**Total**
Lovage	16,4±2,6^{b}	181,4±24,1^{c}	27,6±1,2^{c}	123,2±3,3^{d}	4,3±0,1^{a}	588,6±57,2^{b}	nd	59±4,8	nd	**1000,5**
Basil	4,06±0,9^{a}	174,2±9,2^{c}	28,6±0,8^{c}	22,05±2,6^{b}	10,9±0,6^{b}	4,2±0,2^{a}	11,7±2,7^{a}	nd	15,7±0,6^{a}	**271,4**
Oregano	4,11±0,5^{a}	60,3±0,7^{b}	18,4±0,9^{b}	8,5±0,5^{a}	nd	nd	363,3±25,1^{c}	nd	37,2±2,5^{c}	**491,8**
Thyme	nd	12,06±0,5a	11,4±0,6^{a}	26,03±0,6^{c}	nd	nd	202,3±0,1^{b}	nd	28,06±1,4^{b}	**279,85**

NCA – neochlorogenic acid, CA - caffeic acid, p-CA -p-coumaric acid, FA – ferulic acid, MYR – myricetin, QUE – quercetin, LUT – luteolin, KEMP – kaempferol, API – apigenin; nd – not detected

It was according to the methodology: Wojdyło, A.; Oszmiański, J.; Czemerys, R. Antioxidant activity and phenolic compounds in 32 selected herbs. Food Chemistry, 2007,105, 940-949.

Table S2. Mean scores of consumer desirability of extruded snacks with fermented broad bean or bean and herb/spices, and the control sample (without herb/spices addition).

		Desirability				
		aroma	colour	taste	texture	overall
broad bean grinded	Control	5.21±0.9[a]	6.51±0.8[a]	4.49±1.0[a]	7.25±0.7[a]	4.49±1.1[a]
	Thyme	4.96±1.2[a]	6.11±0.9[a]	4.58±0.9[a]	7.15±0.5[a]	4.58±0.9[a]
	Oregano	4.93±1.3[a]	6.27±0.7[a]	4.93±0.8[a]	7.19±0.9[a]	4.93±1.0[a]
	Basil	5.78±1.1[a]	6.18±0.8[a]	4.05±1.0[a]	7.16±1.0[a]	4.04±1.0[a]
	Lovage	4.96±1.2[a]	6.26±1.1[a]	7.98±0.7[b]	7.16±1.1[a]	7.92±0.8[b]
broad bean husk	Control	3.80±0.8[a]	5.50±0.9[a]	4.15±0.9[a]	7.09±0.9[a]	4.15±1.1[a]
	Thyme	4.72±1.1[a]	5.70±0,9 [a]	4.25±0.8[a]	7.01±0.7[a]	4.25±1.2[a]
	Oregano	4.74±1.0[a]	5.74±0.7 [a]	4.62±0.9[a]	7.06±0.7[a]	4.59±1.0[a]
	Basil	5.32±1.1[a]	5.79±1.2[a]	4.01±1.1[a]	7.03±0.8[a]	3.99±1.3[a]
	Lovage	4.85±1.2[a]	5.85±1.2[a]	7.55±0.9[b]	7.03±1.1[a]	7.75±0.7[b]
bean e grinded	Control	4.15±0.8[a]	5.50±1.0[a]	4.53±1.0[a]	7.09±1.0[a]	4.53±1.0[a]
	Thyme	4.72±1.1[a]	5.70±1.1[a]	4.35±0.9[a]	7.01±1.1[a]	4.35±1.1[a]
	Oregano	4.75±1.1[a]	5.74±1.2[a]	4.72±1.2[a]	7.06±0.9[a]	4.72±1.0[a]
	Basil	5.33±1.2[a]	5.79±1.0[a]	3.75±0.9[a]	7.03±1.2[a]	3.90±0.9[a]
	Lovage	4.85±1.1[a]	5.85±1.2[a]	7.75±1.0[b]	7.03±1.1[a]	7.95±0.8[b]
bean husk	Control	3.93±0.9[a]	4.82±1.2[a]	4.29±0.9[a]	6.93±0.9[a]	4.29±0.9[a]
	Thyme	4.68±1.3[a]	5.65±1.3[a]	4.21±1.1[a]	6.90±1.0[a]	4.12±1.1[a]
	Oregano	4.67±1.2[a]	5.69±0.9[a]	4.61±1.0[a]	6.95±1.1[a]	4.43±1.0[a]
	Basil	5.45±1.0[a]	5.73±0.9[a]	4.03±0.9[a]	6.93±0.9[a]	4.06±1.1[a]
	Lovage	4.55±1.1[a]	5.80±0.9[a]	7.68±0.7[b]	6.93±1.1[a]	7.68±0.8[b]

* Different letters within the same column represent significant differences (n=380), at a α≤0.05

Table S3. Mean scores (n=17) of sensory taste and aroma profiling of extruded snacks with fermented broad bean or bean and herb/spices, and the control sample (without herb/spices addition).

Sample		Descriptors															
		aroma								taste							
		essential oil	herbal	starch	lemon	bitter	strange	sour	broth	essential oil	herbal	sour	starch	salty	broth	bitter	strange
Fermented broad bean grinded	Control	0.25 ^{a*}	3.25 ^{cb}	0.00 ^{a}	3.00 ^{c}	2.00 ^{b}	3.25 ^{cb}	0.00 ^{a}	0.85 ^{a}	0.55 ^{a}	0.55 ^{a}	1.00 ^{ba}	3.00 ^{ba}	1.12 ^{a}	0.00 ^{a}	2.50 ^{cb}	3.00
	Thyme	1.50 ^{cb}	3.40 ^{c}	1.00 ^{cb}	3.20 ^{dc}	0.85 ^{ba}	0.00 ^{a}	0.30 ^{ba}	1.30 ^{ba}	1.00 ^{ba}	1.00 ^{ba}	0.50 ^{a}	3.50 ^{ba}	1.65 ^{ba}	0.00 ^{a}	3.50 ^{dc}	1.75 ^{ba}
	Oregano	3.00 ^{dc}	2.50 ^{b}	1.25 ^{c}	2.25 ^{cb}	0.85 ^{ba}	0.00 ^{a}	0.25 ^{ba}	1.85 ^{b}	1.80 ^{b}	1.80 ^{b}	0.45 ^{a}	3.00 ^{ba}	2.01 ^{ba}	0.00 ^{a}	1.75 ^{ba}	1.50 ^{ba}
	Basil	1.00 ^{b}	1.80 ^{ba}	1.00 ^{cb}	1.95 ^{ba}	0.80 ^{ba}	0.00 ^{a}	0.25 ^{ba}	1.25 ^{a}	0.85 ^{ba}	0.85 ^{ba}	0.75 ^{ba}	2.50 ^{a}	2.00 ^{ba}	0.75 ^{b}	4.50 ^{d}	2.25 ^{b}
	Lovage	1.00 ^{b}	1.20 ^{a}	0.75 ^{b}	0.75 ^{a}	0.85 ^{ba}	0.50 ^{ba}	1.95 ^{c}	1.00 ^{a}	0.85 ^{ba}	0.85 ^{ba}	0.75 ^{ba}	4.00 ^{ba}	2.50 ^{ba}	4.50 ^{c}	0.75 ^{a}	0.75 ^{a}
Fermented broad bean husk	Control	0.00 ^{a}	3.00 ^{cb}	0.50 ^{b}	4.00 ^{d}	3.95 ^{c}	2.85 ^{cb}	0.00 ^{ba}	0.95 ^{a}	0.25 ^{a}	0.25 ^{a}	1.15 ^{b}	4.05 ^{ba}	1.25 ^{ba}	0.00 ^{a}	4.75 ^{d}	3.50
	Thyme	1.85 ^{cb}	3.00 ^{cb}	1.00 ^{cb}	2.45 ^{cb}	1.25 ^{ba}	0.00 ^{a}	0.25 ^{ba}	1.25 ^{ba}	1.25 ^{ba}	1.25 ^{b}	0.50 ^{a}	3.50 ^{ba}	1.85 ^{ba}	0.00 ^{a}	3.75 ^{dc}	1.50 ^{ba}
	Oregano	3.10 ^{dc}	2.25 ^{ba}	1.25 ^{c}	2.00 ^{b}	0.55 ^{a}	0.00 ^{a}	0.25 ^{ba}	2.25 ^{b}	2.00 ^{b}	2.00 ^{b}	0.45 ^{a}	3.00 ^{ba}	2.12 ^{ba}	0.00 ^{a}	1.50 ^{ba}	1.80 ^{ba}
	Basil	1.25 ^{b}	2.00 ^{ba}	1.00 ^{c}	2.50 ^{cb}	0.80 ^{ba}	0.00 ^{a}	0.25 ^{ba}	1.00 ^{a}	0.75 ^{ba}	0.75 ^{ba}	0.75 ^{ba}	2.50 ^{a}	1.98 ^{ba}	0.50 ^{b}	4.25 ^{d}	2.00 ^{b}
	Lovage	0.85 ^{ba}	1.25 ^{a}	0.75 ^{b}	0.80 ^{a}	0.70 ^{a}	0.65 ^{ba}	1.25 ^{cb}	1.25 ^{ba}	0.70 ^{a}	0.70 ^{ba}	0.85 ^{ba}	4.50 ^{b}	2.62 ^{a}	3.50 ^{c}	0.80 ^{a}	0.80 ^{a}
Fermented bean grinded	Control	0.30 ^{a}	3.25 ^{cb}	0.00 ^{a}	3.00 ^{c}	1.00 ^{ba}	3.25 ^{cb}	0.00 ^{ba}	0.75 ^{a}	0.40 ^{a}	0.40 ^{a}	1.75 ^{b}	3.00 ^{ba}	1.20 ^{ba}	0.00 ^{a}	3.00 ^{a}	1.75 ^{ba}
	Thyme	1.75 ^{cb}	3.25 ^{cb}	0.80 ^{cb}	3.25 ^{c}	1.00 ^{ba}	0.00 ^{a}	0.25 ^{ba}	1.00 ^{a}	1.00 ^{ba}	1.00 ^{ba}	0.75 ^{ba}	3.45 ^{ba}	1.80 ^{ba}	0.00 ^{a}	3.25 ^{dc}	1.50 ^{ba}
	Oregano	3.25	2.50 ^{b}	1.20	2.00 ^{b}	0.85 ^{ba}	0.00 ^{a}	0.25 ^{ba}	1.75 ^{ba}	1.50 ^{b}	1.50 ^{b}	0.50 ^{a}	2.95 ^{ba}	2.25 ^{ba}	0.20 ^{ba}	1.75 ^{ba}	1.25 ^{ba}
	Basil	0.85 ^{ba}	1.85 ^{ba}	0.85 ^{cb}	1.50 ^{ba}	0.60 ^{a}	0.00 ^{a}	0.35 ^{ba}	1.10 ^{a}	0.90 ^{ba}	0.90 ^{ba}	0.80 ^{ba}	2.45 ^{a}	1.85 ^{ba}	0.75 ^{b}	4.00 ^{d}	2.20 ^{b}
	Lovage	0.75 ^{ba}	1.35 ^{a}	0.70 ^{b}	0.75 ^{a}	0.80 ^{ba}	0.75 ^{b}	2.25 ^{c}	0.85 ^{a}	0.85 ^{ba}	0.85 ^{ba}	0.75 ^{ba}	4.30 ^{ba}	2.59 ^{b}	3.90 ^{c}	0.80 ^{a}	0.80 ^{a}
Fermented bean husk	Control	0.00 ^{a}	3.50 ^{c}	0.25 ^{b}	4.00 ^{d}	3.95 ^{c}	2.85 ^{b}	0.00 ^{ba}	0.95 ^{a}	0.25 ^{a}	0.25 ^{a}	1.00 ^{ba}	3.75 ^{ba}	1.40 ^{ba}	0.00 ^{a}	3.25 ^{dc}	3.25 ^{a}
	Thyme	1.85 ^{cb}	3.25 ^{cb}	0.80 ^{cb}	2.45 ^{cb}	1.25 ^{ba}	0.00 ^{a}	0.25 ^{ba}	1.30 ^{a}	1.25 ^{ba}	1.25 ^{b}	0.65 ^{a}	3.25 ^{ba}	1.56 ^{ba}	0.00 ^{a}	3.50 ^{dc}	1.75 ^{b}
	Oregano	3.10 ^{dc}	2.84	1.20	2.00 ^{b}	0.55 ^{a}	0.00 ^{a}	0.25 ^{ba}	2.25 ^{b}	2.00 ^{b}	2.00 ^{b}	0.45 ^{a}	3.25 ^{ba}	2.30 ^{ba}	0.00 ^{a}	1.50 ^{ba}	1.80 ^{b}
	Basil	1.25 ^{b}	2.00 ^{ba}	0.85 ^{cb}	2.50 ^{cb}	0.80 ^{ba}	0.00 ^{a}	0.25 ^{ba}	1.00 ^{a}	0.75 ^{ba}	0.75 ^{ba}	0.75 ^{ba}	2.50 ^{a}	1.99 ^{ba}	0.50 ^{ba}	4.25 ^{d}	2.00 ^{b}
	Lovage	0.85 ^{ba}	1.25 ^{a}	0.50 ^{b}	0.80 ^{a}	0.70 ^{a}	0.65 ^{b}	1.25 ^{cb}	1.25 ^{a}	0.85 ^{ba}	0.85 ^{ba}	0.85 ^{ba}	4.50 ^{ba}	2.95 ^{b}	3.50 ^{c}	0.80 ^{a}	0.80 ^{a}

* *Different letters within the same column represent significant differences (n=17), at a $\alpha \leq 0.05$
